# Identifying potential predictors of pain–related disability in Turkish patients with chronic temporomandibular disorder pain

**DOI:** 10.1186/1129-2377-14-17

**Published:** 2013-03-14

**Authors:** Meltem Ozdemir-Karatas, Kadriye Peker, Ali Balık, Omer Uysal, Erman B Tuncer

**Affiliations:** 1 Department of Maxillofacial Prosthodontics, Faculty of Dentistry, Istanbul University, Istanbul, Capa, 34093, Turkey; 2 Department of Dental Public Health, Faculty of Dentistry, Istanbul University, Istanbul, Capa, 34093, Turkey; 3 Department of Medical Statistics and Informatics, Medical School, Bezmialem Vakif University, Istanbul, Fatih, 34093, Turkey; 4 Department of Maxillofacial Prosthodontics, Faculty of Dentistry, Istanbul University, Istanbul, Capa, 34093, Turkey

**Keywords:** Temporomandibular disorders, Pain-related impairment, Chronic pain, RDC/TMD, SCL-90-R, Treatment-seeking behavior

## Abstract

**Background:**

The aims of this study were to examine whether patients’ psychosocial profiles influence the location of pain, and to identify the clinical and psychosocial predictors of high levels of pain-related disability in temporomandibular disorders (TMD) patients with chronic pain at least 6 months in duration.

**Methods:**

The Research Diagnostic Criteria of TMD (RDC/TMD) data for Axis I and II were obtained for 104 consecutive patients seeking treatment.

Data were analyzed using descriptive statistics, *t*-test, Mann–Whitney *U*-test, chi-square test, One-way ANOVA, Kruskal-Wallis test, and binary multiple logistic regression tests. Patients were classified into two groups according to Graded Chronic Pain Scale scores: Grade III and IV were scored for patients with high levels of pain-related disability, whereas Grade I and II were scored for patients with low disability.

**Results:**

Muscle and joint pain were found in 64.9% and 31.8% of the patients, respectively, and 27.3% of the patients suffered from both muscle and joint pain.

Psychosocial disability was found in 26% of patients. There were no statistically significant differences among the diagnostic subgroups with regards to the demographic, behavioral, psychological, and psychosocial characteristics. Patients with high levels of pain-related disability had significantly higher depression, somatization, pain intensity and jaw disability scores than those with low levels of pain-related disability.

Patients with high levels of pain-related disability were more likely to have higher pain intensity, to report higher somatization symptoms and functional impairment, and were less likely to have joint pain than those with low levels of pain related disability.

**Conclusion:**

In conclusion, the Turkish version RDC/TMD, based on a dual axis system, may be used to screen chronic TMD patients at high-risk for pain-related disability who need comprehensive care treatment program.

## Background

Temporomandibular disorders (TMD) are now recognized as a group of biopsychosocial illnesses characterized by chronic painful conditions and dysfunction in the muscles of mastication and the temporomandibular joint [1-4]. The cause of TMD is considered multifactorial with physical, psychological and psychosocial factors, alone or in combination, contributing to the predisposition, initiation or perpetuation of TMD [1,2]. In TMD patients with chronic pain conditions, psychological factors are better predictors of treatment outcome than physical findings [5,6]. As a result, treatment philosophies have moved from the traditional mono-disciplinary approach to the multimodal biopsychosocial approach for reducing pain and improving function in TMD patients [1,5-11]. Within biopsychosocial model, the Research Diagnostic Criteria for TMD (RDC/TMD) is the best and mostly preferred classification system that allows for standardization and replication of research focusing on jaw muscle- and temporomandibular joint (TMJ)-related pain and dysfunction [12-14], and for predicting chronicity in acute temporomandibular joint disorders [15-17]. The Graded Chronic Pain Scale [4], which is a measure of pain impact and disability has been used in many studies examining the prevalence of TMD subtypes, psychological distress, and psychosocial dysfunction in patients [18-24]. However, little research has been conducted to identify clinical and psychosocial factors associated with high levels of pain-related impairment in these patients [22,25,26].

Researchers have reported that pain-related disability is associated with widespread pain [27,28], the frequency of headache [29], undesirable life events [30], illness behaviors [31], treatment-seeking behavior [25], depression [22,25,26,32], somatization [22,25,26,32], and pain duration [22,25]. There are many studies investigating the risk factors for dysfunctional TMD pain [27-33] and the relationship between the psychosocial status and pain severity in TMD patients with chronic pain [26,34]. Recent studies have reported that psychological conditions (i.e. depression and catastrophizing) contribute to the progression of chronic TMD pain and disability [32] and that psychosocial variables (i.e., psychosocial symptoms, affective distress, somatic awareness, and pain catastrophizing) may be premorbid risk factors for the development of chronic TMD pain [33].

Previous studies suggested that TMD patients with muscle disorders may present a higher rate of psychosocial impairment than those with articular disorders [23,25,26,35,36]. The estimated prevalence of psychosocial dysfunction in TMD patients varies from 4.2% to 30% depending on the country and clinical setting [4,18,19,21,22,25,26,37],[38].

Recent studies from Turkey showed that jaw pain is the most frequently reported symptom of TMD in adult population [39], while myogenous TMD pain is more common in TMD patients [40]. Only two studies have examined the psychopathological and clinical features in Turkish TMD patients [40,41].

Researchers suggested that further studies are required to improve the integrated knowledge on the relationship between the different axis I and II diagnoses and it may provide valuable information regarding the clinical predictors of high levels of pain-related disability that influence clinical decision-making process [25,42]. To our knowledge, there is only one study determining the relationship between physical findings and disability levels in TMD patients with chronic pain both acute and chronic pain [25].

The aims of this study were, (a) to examine whether patients’ psychosocial profiles influence the location of pain (masticator muscles, the TMJ, or both), and (b) to identify the clinical and psychosocial predictors of high levels of pain-related disability in TMD patients with chronic pain at least 6 months in duration.

## Methods

### Participants and study design

This cross-sectional study was carried out on a series of consecutive patients seeking treatment for the complaints regarding the masticatory muscles and TMJ in the Department of Prosthodontics, Faculty of Dentistry, Istanbul University, between July and September 2012. A total of 104 patients (40 male and 64 female) were selected from 152 consecutive patients who referred to the Department of Prosthodontics. Of the 104 patients who were enrolled, 43 were self-referred and 61 were referred from general and specialist dental or medical practitioners in the community. The inclusion criteria were the presence of a painful TMD as classified according to RDC/TMD, and complaint of pain at the temporomandibular region and/or masticator muscles for at least 6 months in duration. Patients were excluded if they: (1) had no TMD diagnosis based on the RDC/TMD; (2) had acute TMD pain for less than 6 months; (3) had systemic rheumatic disease; (4) were under the age of 18 years; (5) had a history of psychiatric illness; (6) were pregnant; or (7)were unable or unwilling to consent. Accordingly, 48 patients were excluded, and 41 patients did not meet the inclusion criteria (18 had acute TMD pain, 9 had no TMD diagnosis, 5 had a history of psychiatric illness, 2 failed to give informed consent,3 were pregnant, and 4 had systemic rheumatic disease). In addition, 7 patients who had arthralgia, arthritis, or arthrosis were excluded due to small numbers.

The study was approved by the ethics committee of Istanbul Faculty of Medicine and conducted in accordance with the principles of the Helsinki declaration. All subjects were informed about the nature of the study by a clinic assistant (A. B.). Informed consent was obtained from each subject who agreed to participate in the study prior to filling out the questionnaires.

After finishing the questionnaire, all patients were clinically examined by a trained clinic assistant (M. O.K.) in accordance with the RDC/TMD version 1.0 guidelines [12]. A translated Turkish version of RDC/TMD was used in this study which is available on the RDC/TMD consortium website [43].

All participants were subsequently divided into 3 groups based on their RDC/TMD axis I diagnostic groups. These were as follows: group A, myofascial pain only (n = 43); group B, joint pain only due to disk displacement (n = 20); and group C, myofascial and joint pain due to disk displacement (n = 41).

Subjects completed the RDC/TMD Axis II self-report measures, which include the Graded Chronic Pain Scale (GCPS), the Jaw Disability Checklist, and the Revised Symptom Checklist (SCL-90-R).

Functional status was evaluated using the jaw disability checklist that contains 12 items concerning limitations in activities related to mandibular functioning. The answer to each item might be ‘No’ (0)or ‘Yes’(1). Total scores might range from 0 to 12 [12]. Cronbach’s alpha coefficient for this sample was 0.76.

Psychosocial functioning was assessed using the GCPS, which is an indicator of the extent to which pain is perceived by the patient and the degree to which the pain is disabling [4,24]. The GCPS assesses pain intensity, interferences with usual activities, family and leisure activities, work-related activities, and disability days due to pain. Characteristic Pain Intensity (CPI) was evaluated through scoring items in the questionnaire about pain history. The patient’s score ranged from 0 to 100, with 100 being the most intense pain. Cronbach’s alpha of CPI in this study was 0.79.

The GCPS was used to classify individuals according to the chronic pain grades: I = low pain intensity and low levels of pain-related disability, II = high pain intensity and low levels of pain-related disability, III = moderate pain-related disability and IV = severe pain-related disability. The study sample was then split into two groups, based on GCPS scores, which were assumed as the indicators of treatment need. The first group included subjects with high levels of pain-related disability (GCPS grades III and IV), whereas the second group included subjects with low levels of pain-related disability (GCPS grade I and II).

The psychological status was assessed through depression and non-specific physical symptom (somatization) scores measured with subscales of SCL-90-R [12]. The questionnaire included 32 questions with 5-point Likert response scale, 12 items of somatization subscale and 20 items of depression subscale. The mean score ranged from 0 to 4 for both depression and somatization subscales. Cronbach alpha levels for this sample were 0.87 and 0.93 for the somatization and depression scales, respectively.

Patient’s treatment-seeking status before attending Prosthodontics Clinics of Istanbul University was evaluated by a single specific question of the RDC/TMD axis II questionnaire: "Have you ever gone to physician, dentist, chiropractor or other health professional for facial ache or pain?” Then, the patients were classified into two groups according to their response: treatment-seeking group and non-treatment-seeking group.

Additional data related to nonspecific pain condition, general health status perception, pain medication use, education level, employment, and marital status of patients were gathered using the axis II questionnaire and a single-item measure of the frequency of pain medication. These data were not included in the analysis, because it will be used in a future study evaluating the premorbid psychosocial and behavioral risk factors of chronic TMD pain.

### Statistical analysis

Descriptive data on frequencies, proportions, and on means and standard deviations were obtained with respect to the demographic, behavioral, psychological, and psychosocial characteristics of the groups. Subsequently, the normality of the SCL–90-R subscale scores, age, CPI, jaw disability scores and pain duration was checked using Kolmogorov-Smirnov test. Continuous data were analyzed by means of One-way ANOVA and Kruskal-Wallis test, for detection of possible differences between the variables under investigation among the TMD diagnostic subgroups, and categorical variables were analyzed using Pearson's chi-square test.

Patients with high and low levels of pain – related disability were compared with regards to socio-demographic, behavioral, psychological, and psychosocial characteristics using chi-square tests for categorical data, t-tests for continuous parametric data and Mann–Whitney *U*-test for continuous non-parametric data. The internal consistency of the Axis II measures was evaluated using the Cronbach's alpha reliability coefficient. A priori power analysis was performed by using an online calculator (http://statpages.org/index.html#Power) to determine sample size needed for the binary logistic regression analysis. Sample size calculations based on an alpha of 0.05, 10 predictor variables, expected effect size of 0.20 (moderate effect size), and power of 0.80 indicated a required sample size of 91 subjects.

A binary logistic regression analysis with stepwise backward elimination (likelihood ratio) was performed to identify the significant associations between the predictors (independent variables: sex, age, pain duration, CPI, jaw disability score, treatment-seeking behavior, myofascial pain, disk displacement, depression and somatization scores) and the outcome (dependent variable: high levels of pain- related disability). The predictive power of the model was tested using Omnibus Test of Model, and Hosmer and Lemeshow tests were used to examine goodness of the fit of the model. Nagelkerke R square was generated to express the proportion of variance explained by the model. The odds ratio (OR) and 95% confidence intervals (95% CI) were calculated using binary logistic regression models. Statistical significance was achieved when p < 0.05. Statistical analysis was performed using IBM SPSS Statistics 19 for Windows (SPSS Inc., Chicago, IL, USA).

## Results

The study sample consisted of 104 patients (61.5% females, 38.5% males) with a mean age of 33.46 ± 10.51 years. The mean chronic pain duration at the time of the assessment was 25.90 ± 28.76 months (range 7–168 months). Fifty-nine percent of these patients reported treatment-seeking for TMD pain before referring to the Prosthodontics Clinics of Istanbul University. From 104 patients, 51% (n = 53) were single, 48.1% (n = 50) had a formal school education of less than or equal to 8 years, 54.8% (n = 57) were employed, 32.7% (n = 34) rated their health status as moderate, and 87.5% (n = 91) reported a recent history of headache or pain in the lower back, chest/heart, or nausea/abdominal pain (data not shown).

The frequencies of the TMD diagnostic subgroups were as follows: group A, 41.3%; group B, 19.2%; and group C, 39.4%. There were no statistically significant differences among the TMD diagnostic subgroups with regards to the demographic, behavioral, psychological, and psychosocial characteristics as seen in Table 1.

**Table 1:** **Demographic**, **behavioral**, **psychological**, **and psychosocial characteristics of the 104 TMD patients with chronic pain**, **according to pain location**

**Characteristics**	**RDC**/**TMD subgroups**			**p value**
	**Group A (43)**	**Group B (20)**	**Group C (41)**	
**Gender**^**a**^				
Male	22 (51.2%)	6 (30%)	12 (29.3%)	0.082
Female	21 (48.8%)	14 (70%)	29 (70.7%)	
**Depression **^**b**^ (Mean ± SD)	1.57 ± 0.89	1.13 ± 0.87	1.45 ± 0.95	0.213
**Somatization **^b^ (Mean ± SD)	1.64 ± 0.81	1.25 ± 0.90	1.79 ± 0.95	0.085
**Pain duration **^c^ (Mean ± SD)	20.67 ± 20.50	33.55 ± 44.31	27.65 ± 26.33	0.446
Jaw disability checklist ^b^ (Mean ± SD)	5.25 ± 2.70	5.40 ± 2.50	5.60 ± 3.02	0.845
**CPI **^**b**^ (Mean ± SD)	63.10 ± 19.27	59.50 ± 16.62	66.34 ± 20.55	0.420
**Age **^**b**^ (Mean ± SD)	30.69 ± 9.58	35.80 ± 10.30	35.21 ± 11.11	0.077
**Treatment**-**seeking**^**a**^				
No	19 (44.2%)	8 (40%)	16 (39%)	0.883
Yes	24 (55.8%)	12 (60%)	25 (61%)	
**Disability**^**a**^				
High	12 (46.2)	5 (19.2)	9 (34.6)	0.820
Low	31 (39.7)	15 (19.2)	32 (41)	

SD, standard deviation; CPI, Characteristic Pain Intensity; Group A, myofascial pain only; Group B, joint pain only due to disk displacement; Group C, myofascial and joint pain due to disk displacement.

^a^ Statistical evaluation by the chi-square test.

^b^Statistical evaluation by *t* test.

^c^Statistical evaluation by Mann–Whitney *U* test.

Based on GCPS scores, psychosocial disability (grade III or IV) was revealed in 26% of TMD patients.

Bivariate analysis showed that patients with high levels of pain-related disability had significantly higher depression, somatization, CPI and jaw disability scores than patients with low levels of pain-related disability. No significant differences were found between patients with high and low levels of pain-related disability in terms of age, gender, RDC/TMD diagnostic sub- groups, and pain duration. Patients who had never been treated before referring to our clinic reported lower levels of pain–related disability (Table 2).

**Table 2:** **Frequency distribution of patients**' **pain**- **related disability according to demographic**, **behavioral**, **psychological**, **and physical characteristics**

	**Low**	**High**	
**Characteristics**	**n (%)**	**n (%)**	**p value**
**Gender**^**a**^			
Male	28 (35.9%)	12 (46.2%)	0.352
Female	50 (64.1%)	14 (53.8%)	
**Treatment**-**seeking**^**a**^			
No	38 (48.7%)	5 (19.2%)	0.011
Yes	40 (51.3%)	21 (80.8%)	
**RDC**^**a**^			
Muscle	31 (39.7%)	12 (46.2%)	0.820
Disc	15 (19.2%)	5 (19.2%)	
Muscle&disc	32 (41%)	9 (34.6%)	
**Depression **^**b**^ (Mean ± SD)	1.29 ± 0.86	1.89 ± 0.92	0.003
**Somatization **^**b**^ (Mean ± SD)	1.47 ± 0.93	2.08 ± 0.58	0.000
**Age **^**b**^ (Mean ± SD)	32.78 ± 11.12	35.50 ± 8.27	0.256
**Pain duration **^**c**^ (Mean ± SD)	25.97 ± 30.22	25.69 ± 24.38	0.258
**Jaw disability checklist **^**b**^ (Mean ± SD)	4.69 ± 2.44	7.61 ± 2.60	0.000
**CPI **^**b**^ (Mean ± SD)	57.90 ± 18.21	81.02 ± 9.92	0.000

SD, standard deviation; CPI, Characteristic Pain Intensity.

^a^ Statistical evaluation by the chi- square test.

^b^Statistical evaluation by *t* test.

^c^Statistical evaluation by Mann–Whitney *U* test.

Stepwise binary logistic regression analyses were performed to examine the association of the variables under investigation with higher levels of pain- related disability. In the final model, only four variables were found to be associated with higher levels of pain-related disability after removing non-significant variables. The final model had a statistically significant predictive power (*χ*^2^ = 61.745, p < 0.0001; Hosmer and Lemeshow goodness of fit *χ*^2^ = 6.910, p = 0.546) and overall correctly classified 83.7% of the patients.

The final model explained 66% of the variance for the level of pain-related disability of patients with chronic TMD pain (Nagelkerke's R square = 66.3%; Table 3). Patients with high levels of pain-related disability were more likely to have higher pain intensity (OR = 1.16, 95% CI = 1.07-1.27), and to report higher somatization symptoms (OR = 3.66, 95% CI = 1.16-11.49) and mandibular functional impairment (OR = 1.63, 95% CI = 1.18-2.23), and were less likely to have joint pain (OR = 0.22, 95% CI = 0.06-0.73), than those with low levels of pain related disability (Table 3).

**Table 3:** **Predictors of high pain**-**related disability**

**Significant predictors**	**OR** (**95**% **CI**)	**p value**
**Somatization**^**b**^	3.66 (1.16-11.49)	0.026
**Jaw disability checklist **^**b**^	1.63 (1.18-2.23)	0.002
**CPI**^**b**^	1.16 (1.07-1.27)	0.000
**Joint pain**^**a**^	0.22 (0.06-0.73)	0.014

^a^ Dichotomized variables: Joint pain (0 = having joint pain,1 = no having joint pain).

^b^Continous variables: Somatization was measured by the 12- item somatization subscale of the of SCL-90-R; Jaw disability checklist which contains 12 items concerning limitations in activities related to mandibular functioning; CPI was evaluated through scoring items in the questionnaire about pain history. The patient’s score ranges from 0 to 100, with 100 being in the most pain.

CI, confidence interval; OR, odds ratio.

## Discussion

Recent studies have shown that psychosocial and behavioral factors are important predictors of treatment outcome [42,43]. Thus, a multidimensional and biopsychosocial approach is recommended to be used for the assessment and management of TMD patients with major psychosocial involvement which determines a combination of biological, psychological, behavioral and social factors [1,5-11].

To date, only one multicenter study explored the relationship between physical findings and disability levels in TMD patients with acute and chronic pain [25]. To our knowledge, this is the first study to examine the relationship between RDC/TMD axis I and axis II findings and to identify the predictors of high levels of pain-related disability in patients with chronic TMD pain. In this study, RDC/TMD was used because it is currently the most widely accepted method for assessing both physical and biobehavioral aspects of TMD. In this study, the temporal criterion (pain lasting for at least 6 months) was used to select the TMD patients suffering from chronic pain, as well as in many others [4,6,22,26]. Patients were classified into two groups based on GCPS scores: the first group included subjects with high levels of pain-related disability and and the second included subjects with low levels of pain-related disability. Manfredini et al. [44] reported that the factor “time since pain onset” is not likely to be the most important predictor of pain-related disability. Von Korff and Dunn [45] indicated that a prognostic risk score had better predictive validity for pain outcomes than did the pain duration alone for back pain, headache, and orofacial pain. In contrast to duration – based defining, defining chronic pain prospectively, by risk thresholds for future clinically significant pain accepts that chronic pain has multiple attributes, including psychological and behavioral components, in addition to pain severity and duration [45,46].

Due to the long lead time required to complete the Risk Score questions, we did not calculate patients’ Risk Scores in the present study [45]. In addition to the temporal criterion, we used other important pain characteristics such as the onset of chronic pain, the CPI, treatment-seeking behavior, the impact of pain on patient’s emotional and jaw functional status in accordance with the new definition of chronic pain.

Consistent with previous studies [18,19,25,40], we found that myofascial pain diagnosis was the most common axis I finding. The present findings confirm previous suggestion that the original RDC/TMD facilitate establishing myofascial pain diagnoses [47].

In this study, 35% of patients received multiple axis I diagnoses. This is in line with a previous study [25,38] suggesting that the prevalence of combined muscle and joint disorders is a clinically important reality.

This study showed that the estimated prevalence of psychosocial dysfunction in Turkish patients with chronic TMD pain was higher than that reported in the previous studies on TMD patients [4,18,19,21,22,25,26,37],[38]. This could be explained by the fact that many patients with chronic TMD pain are referred by a dentist or a physician to specialized clinics, such as prosthodontic outpatient clinics or physical medicine and rehabilitation clinics at university hospitals.

We determined that the psychological factors (depression, somatization), pain characteristics (CPI, pain duration), demographic factors (age, gender), treatment-seeking behavior, pain-related impairment, jaw functional limitation were not associated with a specific pain location. Our results are inconsistent with a previous study reporting differences in pain intensity among RDC/TMD Axis I diagnoses [17]. This study confirmed the findings of Reissmann et al. [23] that the location of pain in TMD patients is not a major factor for the prediction of psychosocial profiles and the current pain severity-measured by CPI.

Manfredini et al. [44] reported that there is a close association between pain and psychosocial disorders in TMD patients because of the trend in evidencing higher SCL-90-R scores for myofascial pain patients, alone or combined with TMJ pain compared with TMJ pain alone. The discrepancy between our results and those of Manfredini et al. [44] might be explained by the fact that they did not take into account the pain chronicity as the main study’s variable. Celić et al. [34] reported that the chronic TMD patients and patients with multiple TMD diagnoses had higher rates of depression and somatization, which is in contrast with our findings. Our results were consistent with the study by Kino et al. [48] that also found no differences in functional difficulties across the RDC/TMD diagnostic groups using a newly devised pain-related ‘Limitation of Daily Functions’ for the TMD questionnaire. In contrast to the results of our study, Reissmann et al. [23] reported that jaw function is affected more by joint pain than by myogenous pain.

Bivariate analysis revealed significant differences between patients with low and high levels of pain-related disability in depression, somatization, CPI and jaw disability scores as well as in treatment-seeking behavior. We found no significant differences between patients with high and low levels of pain-related disability in terms of age, gender, RDC/TMD diagnostic sub-groups, and pain duration. In contrast with the results of our study, Maixner et al. [49] reported that younger age and female sex are associated with an elevated risk of first-onset TMD and increased odds of chronic TMD.

The relatively small number of studies examining the relationship between depression and somatization levels in pain-related impairment. In previous studies, pain-related disability was found to be strongly related with depression and somatization levels [13,22] and pain duration [22]. We determined very similar results, except for the finding that pain-related disability was not significantly related with pain duration. Our study confirmed the results of previous studies showing that the dysfunctional pain patients were more likely to be depressed [1,4,22,25,32,34], to report non-specific physical symptoms [1,4,22,24-26,33,50] and higher pain severity ratings [26], and to use more health care [1,25,37] than functional pain patients.

Multivariate analysis showed that patients with high levels of pain- related disability were more likely to have higher pain intensity, to report higher somatization symptoms and mandibular functional impairment, and were less likely to have joint pain due to disk displacement than those with low levels of pain related disability. Consistent with our study, Manfredini et al. [25] found that disk displacement with reduction was a negative predictor of high disability. In contrast to our findings, they reported that predictors for high disability were related to severe depression and somatization as well as psychosocial aspects (pain lasting from more than 6 months; treatment-seeking behavior). This difference between their findings and those in our study may be due to the study design and the differences in patient inclusion criteria. Their study aimed to describe the relationship of RDC/TMD axis I diagnoses with GCPS ratings and to identify predictors of disability levels in a sample comprising both clinic and community subjects. The present study aimed to identify clinical and psychosocial predictors of high levels of pain-related disability in TMD patients who had chronic pain in the masticatory muscles, TMJ, or both. Patients with a diagnosis of arthralgia, arthritis, or arthrosis were not included in this study due to the small number of patients. These patients are generally referred by a primary care physician or a dentist to the Multidisciplinary Temporomandibular Joint Diagnosis and Management Unit at Faculty of Medicine, Istanbul University. In this study, somatization was found to be a significant predictor for high disability, consistent with previous studies of TMD patients with chronic pain [26,51]. This may be explained by the fact that somatization is related to a more widely dispersed pain [13,24,49,52] and nonspecific pain conditions [27], which are highly associated with risk of developing pain-related disability [27,28]. This study’s observed relationship between somatization and pain- related disability may suggest that those two parameters are actually the most clinically valid components of the RDC/TMD axis II psychosocial assessment. More research will be needed to confirm these findings.

We found a strong relationship between pain–related disability and jaw disability. In accordance with this finding, Kafas and Leeson [8] reported that the chronic pain affects patients’ eating and chewing functions. In contrast, a previous study [20] found no significant difference in limitations related to mandibular functioning scores among patients with different graded chronic pain severity classification. Dougall et al. [17] reported that patients' high-risk versus low-risk status for potentially developing chronic TMD significantly affected chewing performance.

It should be noted that we did not find pain duration to be a predictor of high disability in patients with chronic TMD pain, while the duration of pain greater than 6 months was found to be the most important predictor for high disability in TMD patients [22,25]. We found that CPI was a highly significant predictor of high disability in patients with chronic TMD while previous studies using the “high-risk versus low-risk” model for the development of chronic TMD showed that patients with high risk reported more pain on the CPI relatively, than the patients with low risk [15-17]. Future studies are needed to confirm our findings in chronic TMD patients.

Manfredini et al. [25] found that treatment-seeking behavior was a significantly predictor of high disability in TMD patients. In contrast, we did not find that treatment-seeking was a predictor of high disability. Studies suggested that profiling patients' clinical presentations and psychological status can be useful for a better understanding of the treatment-seeking behavior of TMD and for providing appropriate treatment planning [8,9,11,16,20,23,25,26],[44,53]. Additional studies are needed for determining the clinical and psychological factors related to treatment-seeking behaviors in TMD patients with chronic pain.

To the best of our knowledge, this is the first study using “high-risk versus low-risk” model to identify clinical and psychosocial predictors of pain-related disability in patients with chronic TMD pain. Each measures of the translated Turkish version of the RDC/TMD Axis II Questionnaire showed satisfactory internal consistency. There are several limitations to this cross-sectional study that should be considered when interpreting these findings. The study was conducted in one of the three dental faculties in Istanbul, limiting the generalizability of the results and the conclusions. Regarding the health – care seeking behavior, patients who attend a tertiary hospital clinic are probably not representatives of their source population that has the same condition and do not seek any treatment. Therefore, future community – based studies are needed to increase representativeness of groups investigated and generalizability of the findings [54]. Patients with a diagnosis of arthralgia, arthritis, or arthrosis were not included in this study due to the small number of patients. Further multicenter studies are needed to evaluate the potential factors related to pain related disability in different RDC/TMD subgroups. The cross -sectional design did not allow causation or changes over time in pain-related impairment of TMD patients with chronic pain. Future research should focus on longitudinal studies in an attempt to clarify causal relationships between pain – related impairment and chronic pain conditions in TMD patients. Further studies are required to assess the predictive validity of a multifactorial risk score for TMD pain that is based on the pain intensity, interference, and activity limitation day ratings from the GCPS; SCL-90-R depression scale; pain days in the last six months; and the number of pain conditions at sites other than the pain condition index [45,46].

Additional studies are needed to assess the premorbid psychosocial risk factors (i.e., somatic awareness, anxiety, affective distress, psychosocial stress, pain coping and catastrophizing) for chronic TMD pain that may affect the treatment plan in Turkish TMD patients [8,30-33,36,49]. We analyzed our findings using mean scale scores instead of the published cutoff points for patients on both the somatization and depression subscale. Further studies are required to assess both the clinical utility and validity of the depression and somatization subscales of RDC/TMD Axis II using previously validated reference standards [13,14].

## Conclusion

In conclusion, the results of this study emphasize the importance of obtaining both Axis I and Axis II assessments for identifying TMD patients at a high risk for pain- related disability who are characterized by the absence of joint pain, and by increased pain intensity, jaw disability, and somatic symptoms. In our clinic, TMD patients were treated with intra-oral appliances and/or occlusal therapy without considering the patient's psychosocial status. If treatment is unsuccessful, patients are primarily referred to the Multidisciplinary Temporomandibular Joint Diagnosis and Management Unit at Faculty of Medicine, Istanbul University. The Turkish version of a dual-axis system for the diagnosis of TMD may be used by clinicians to screen high-risk patients for pain- related disability who need comprehensive treatment care program.

## Competing interest

The authors declare having no competing financial interest/funding for this paper.

## Authors’ contributions

MOK, KP, AB and EBT were principal investigators of this study. They contributed to the interpretation of the data and review of the manuscript. MOK and KP conceptualized the study, contributed to the interpretation of the data and wrote the manuscript. EBT participated in study design. AB contributed to the data collection. OU contributed to the study design and data analysis and calculated the required sample size. All authors read and approved the final manuscript.
